# Supplementary material for: Deciphering the Monilinia fructicola Genome to Discover Effector Genes Possibly Involved in Virulence
Source: Genes (Basel). 2021 Apr 14;12(4):568. doi: 10.3390/genes12040568 (PMC8070815; doi:10.3390/genes12040568)
Supplement: Supplementary file 1 [file genes-12-00568-s001.pdf]

Supplementary Tables

Supplementary Table 1. Primer design for amplification of the CDS of *Monilinia fructicola* genes.

	Forward primer	Reverse primer
Mf1	CACACA**CTCGAG**GATGGAAACTTTCCTGCTCCCG	CACACA**CTGCAG**TCAAACTACCAAGCATAGTCCTGAAATCG
Mf3	CACACA**CTCGAG**TCTCCTCATAGTCTACCAGTTGAAGCTC	CACACA**CTGCAG**TTAGTAGCCACAAGCCTTCTGTTCTC
Mf7	CACACA**GTCGAC**CTCCCAACCAATGGCGGCAC	CACACA**CTGCAG**TTACAAAATGTTAAGAGGCAAAGAGAG
Mf8	CACACA**GTCGAC**GCTCCCACTTCCTCCCCCGC	CACACA**CTGCAG**CTAAGGGGTCTTTGCCTTCCAAG
Mf9	CACACA**CTCGAG**CGTCCCGCTATCCCCG	CACACA**CTGCAG**CTATGCAGGTGGTAATGGGCAAG
Mf11	CACACA**CTCGAG**ACCCGCGACCTCGCCACCTC	CACACA**ATGCAT**CTACTTGCAGTCACTGCGACCG
MfNEP2	CACACA**GTCGAC**GCGCCATCTCAAATCGAGTCTC	CACACA**ATGCAT**CTAGAAAGTAGCCTTGCCAAGGTTG
